# Repeated Impact-Based Capture of a Spinning Object by a Dual-Arm Space Robot

**DOI:** 10.3389/frobt.2018.00115

**Published:** 2018-10-16

**Authors:** Kenji Nagaoka, Ryota Kameoka, Kazuya Yoshida

**Affiliations:** Department of Aerospace Engineering, Tohoku University, Sendai, Japan

**Keywords:** dual-arm space robot, repeated impact, detumbling of space debris, capture of space debris, motion tracking control, experimental verification

## Abstract

This paper presents detumbling and capture of space debris by a dual-arm space robot for active space debris removal missions. Space debris, such as a malfunctioning satellite or a rocket upper stage, often has uncontrolled tumbling motion. It also has uncertainties in its parameters, such as inertial characteristics or surface frictional roughness. These factors make the debris capture missions difficult to accomplish. To cope with such challenging missions, we propose a detumbling and capture control method for a dual-arm robot based on repeated impact capable of suppressing the debris motion by repeatedly utilizing an effect of a passive damping factor in the contact characteristics. In this paper, as the initial step of a study on the repeated impact-based capture method, we assume that the capture target is a rocket upper stage that can be simply modeled as a cylindrical body and mainly has angular velocity motion in its principle axis of inertia. A motion tracking control law of an end-effector of the robot arm is introduced to maintain the repeated impact. The proposed control method enables the robot to accomplish the detumbling and capture without precise estimation of the inertial characteristics and surface frictional roughness of the debris. The validity of the proposed method is presented by numerical simulations and planar microgravity experiments using an air-floating system. In particular, the experimental evaluation shows the fundamental feasibility of the proposed method, and thus, the result contributes to a practical application.

## 1. Introduction

An active space debris removal system is a critical technology for sustainable utilization of an orbital environment (Liou and Johnson, 2006). In 1992, a manned mission to retrieve a satellite was performed by the extra vehicular activities (EVAs) of astronauts (McBarron II, 1994). Such EVAs for debris removal, however, carry a high risk to astronauts because space debris, such as a malfunctioning satellite or an upper stage booster of rockets, has mostly uncontrolled tumbling motion and uncertainties in its inertial parameters. To achieve secure capture and de-orbit of the space debris, robotic removal missions have been proposed toward practical space debris mitigation. Such an on-orbit servicing robot is generally defined as a satellite with multi-degrees-of-freedom (multi-DOF) robotic arms. In particular, as one of the key technical challenges, robotic detumbling techniques have thus far been addressed for secure capture of an uncooperative target. A net-capture method of tumbling debris has been proposed (Wormnes et al., 2013; Cerceos et al., 2014). Although net capture enables the capture of uncertain debris, it is non-reusable and basically premises preliminary detumbling for secure capture. A repeated impulse-based detumbling method has been proposed by using projectiles (Kitamoto et al., 2002) or robot arms (Yoshikawa and Yamada, 2007). In addition, a contactless detumbling method by inducing eddy currents has been proposed (Kadaba and Naishadham, 1995; Sugai et al., 2013; Gomez and Walker, 2015). However, these methods targeted a fixed-base (not free-flying) chaser system or focused only on the target's spin. Therefore, the relative motion control between the space robot and target during detumbling has not been compensated by these methods, and they do not cover completion of the capture. A rotational motion damper for detumbling with relative motion has been proposed (Matsunaga et al., 2001). This method basically assumes the condition that the target parameters, including mass and moment of inertia, are fully known.

**Figure 1:**
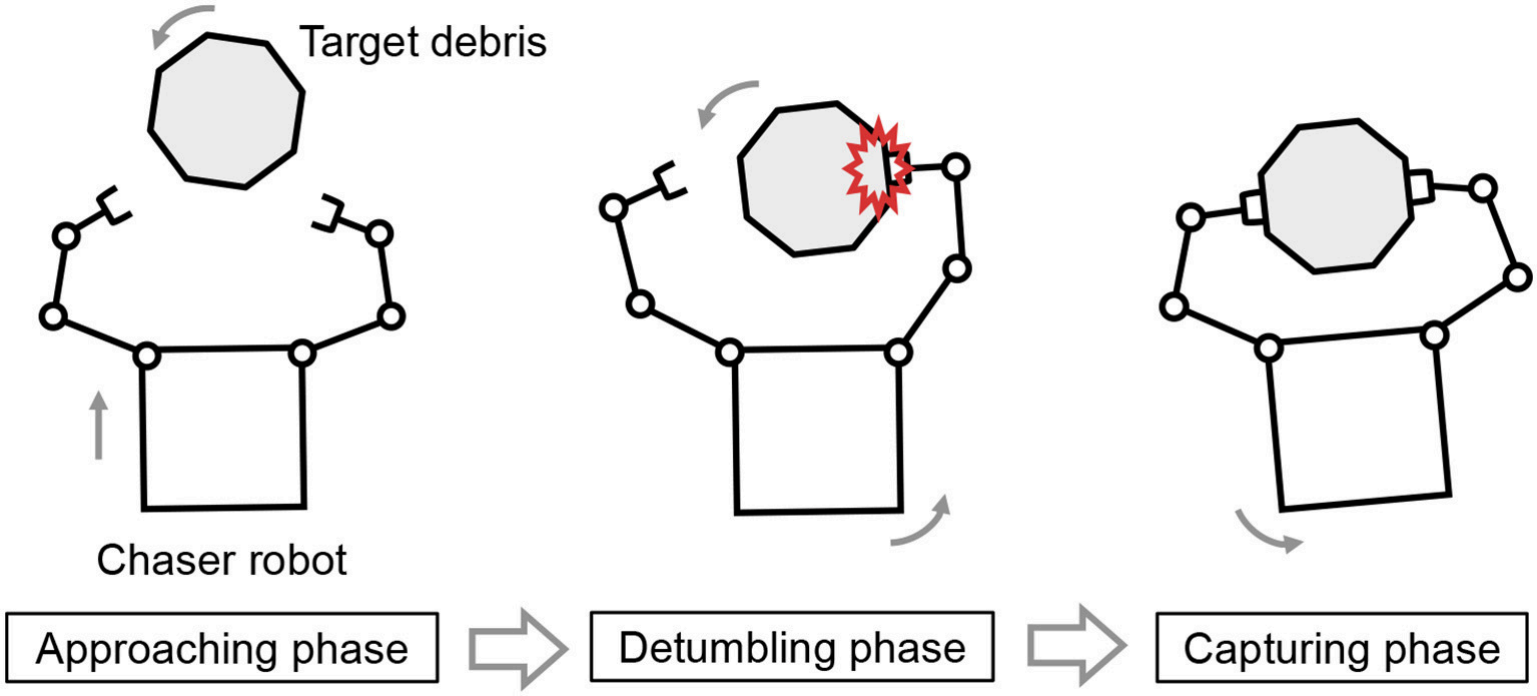
Capture sequence of space debris by a dual-arm space robot.

On the other hand, the detumbling and capture of space debris by a dual-arm robot system has been proposed. Figure 1 illustrates a typical capture sequence of space debris by a dual-arm space robot. The capture sequence is divided into three phases of approaching, detumbling, and capturing. The capture strategy by a dual-arm robot can potentially allow unexpected pushing-away of the debris by impounding of the dual arm unlike a single-arm robot. That is, the dual-arm robot can enclose the target motion with uncertainties by dual arms, whereas the single-arm robot is risk for unexpected pushing-away or collision of uncertain debris without accurate parameter estimation. This study focuses on the robot manipulation in the detumbling and capturing phases after the approaching phase.

As related work, detumbling and capture of a spinning target was experimentally demonstrated by a dual-arm robot based on a hybrid simulator (Takahashi et al., 2008). Although this method achieved both detumbling and capture by a continuous control framework, it requires tracking of the target spin and controlling contact forces. A detumbling control method of a dual-arm space robot based on the impedance control of the space robot (Nakanishi and Yoshida, 2006; Uyama et al., 2012) has also been proposed (Stolfia et al., 2017). Its dual arm contacts the same plane of the uncooperative target. These past works assume that mass and moment of inertia of the target are fully known. Therefore, detumbling and capture of uncertain tumbling debris by a sequential or continuous control framework is a challenging technology. In addition, detumbling and capture technologies associated with experimental validation have been limited. In this paper, we propose a repeated impact-based detumbling and capture method by a dual-arm space robot. The tumbling or spinning motion decays by a repeated damping effect of the repeated impact. In the proposed method, the robot can maintain the repeated impact by which the direction of the target's linear velocity is constrained by controlling each contact point. That is, the relative translational velocity between the robot base and target is zero and each impact imparts a small relative velocity. Moreover, this is limited by a proper choice of the subsequent impact points Therefore, it enables the detumbling and capture of space debris without precise control of contact force depending on target's inertial parameters. The validity of the proposed method is also evaluated by both numerical simulation and a ground experiment that emulates planar microgravity.

This paper is organized as follows. Section 2 introduces definition of the capture target and modeling of the dual-arm space robot with contact dynamics. Section 3 presents a repeated impact-based control law for detumbling the target spin. The control method includes motion tracking control to achieve the repeated impact. The capture sequence is also described. Section 4 shows simulation analysis of the proposed control method. Section 5 shows experimental validation of the proposed control method. A planar microgravity experiment is performed by using an air floating system. The experimental result confirm the fundamental effectiveness and feasibility of the proposed method. Finally, section 6 summarizes the contributions of this paper.

## 2. Modeling

Definition of the capture target and fundamental modeling of the dual-arm space robot and target are presented in this section, prior to discussion of the control law. For generalization, the models are given as three-dimensional.

### 2.1. Capture target

In this paper, to primarily demonstrate an effectiveness of an idea of the repeated impact-based capture, we assume that the capture target is a rocket upper stage as an initial step. The upper stage is one of the space debris to be removed (Williams and Meadows, 1978; Shan et al., 2016; Bylard et al., 2017). Most of the upper stages can be approximately modeled as a cylindrical body, and some of them are also deemed to be a stable floating state with a single spin in its principle axis of inertia because of its mass distribution and gravity from the earth's gravitational attraction. Therefore, this paper focuses on the capture of a cylindrical free-floating object, which can be approximately defined as a planar motion.

### 2.2. Preliminaries

**Figure 2:**
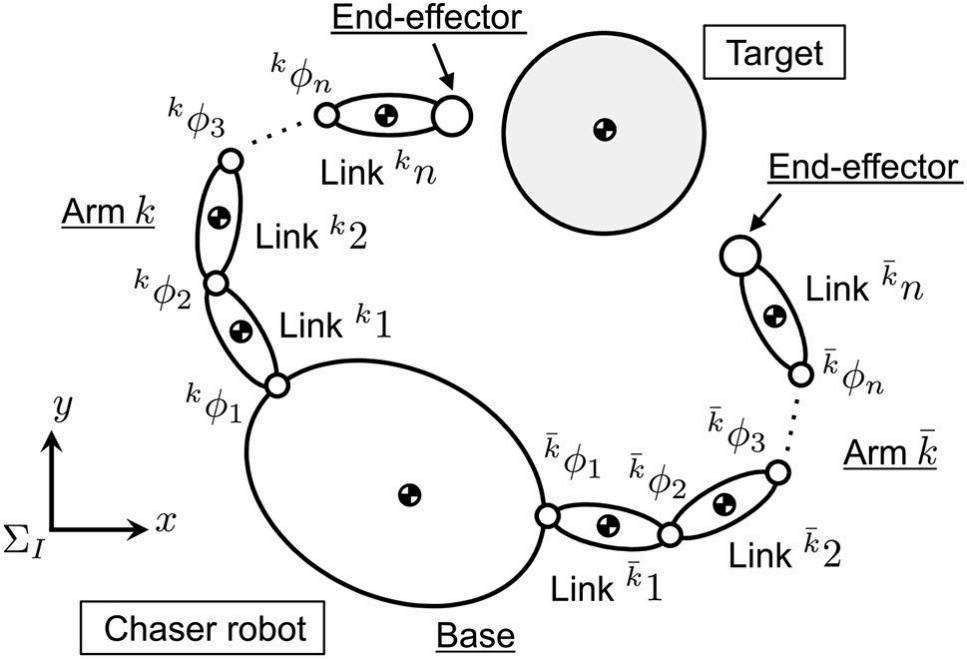
Target capture model by a dual-arm space robot.

Figure 2 illustrates a planar model of a dual-arm robot (chaser robot) and target debris, where Σ_*I*_ is an inertial coordinate system. Prior to the modeling, the following assumptions are defined.

- The influence of gravity acceleration can be ignored because of microgravity in orbit.
- Planar motion is targeted for simplicity.
- The chaser robot is comprised of main base and serial link arms (dual-arm).
- The chaser base, arm's link, and joint, and target are rigid.
- Contact occurs between the arm's end-effector (spherical tip) and target surface.
- The target is a circular object in two dimensions and its center of mass coincides with its geometric center.
- Radius and center of mass position of the target are known.
- Mass, moment of inertia, and frictional property of the target are unknown.
- Contact is point contact, and contact force and torque are generated at only the contact point.
- Contact surface of the target is smooth and uniform.

Here, the radius and center of mass position of the target can also be assumed to be estimated based on an on-orbit measurement of the target's spinning motion.

### 2.3. Equation of motion of dual-arm robot

In this paper, the dual-arm is defined as left and right arms. A superscript *k*, which is 1 or 2, is shown to discern each arm. The link number of each arm is ^*k*^*n* and the whole joint number *n* is *n* =^1^*n*+^2^*n*. The variables are defined in Σ_*I*_, unless otherwise noted. Based on the Lagrange's equation, the equation of motion of the dual-arm space robot is introduced as follows (Umetani and Yoshida, 1993):

(1) $$\begin{array}{l} \left[\begin{array}{ccc} H_{b} & {}^{k}H_{bm} & {}^{\bar{k}}H_{bm}\\ {}^{k}H_{bm}^{T} & {}^{k}H_{m} & 0_{{}^{k}n\times^{\bar{k}}n}\\ {}^{\bar{k}}H_{bm}^{T} & 0_{{}^{k}n\times^{\bar{k}}n}^{T} & {}^{\bar{k}}H_{m} \end{array}\right]\left[\begin{array}{c} {\overset{..}{x}}_{b}\\ {}^{k}\overset{..}{\phi }\\ {}^{\bar{k}}\overset{..}{\phi } \end{array}\right]+\left[\begin{array}{c} c_{b}\\ {}^{k}c_{m}\\ {}^{\bar{k}}c_{m} \end{array}\right]=\left[\begin{array}{c} F_{b}\\ {}^{k}\tau \\ {}^{\bar{k}}\tau \end{array}\right]\\ \;+\left[\begin{array}{cc} {}^{k}J_{b}^{T} & {}^{\bar{k}}J_{b}^{T}\\ {}^{k}J_{m}^{T} & 0_{6\times^{k}n}^{T}\\ 0_{6\times^{\bar{k}}n}^{T} & {}^{\bar{k}}J_{m}^{T} \end{array}\right]\left[\begin{array}{c} {}^{k}F_{h}\\ {}^{\bar{k}}F_{h} \end{array}\right] \end{array}$$

where,

- $\bar{k}$: the other value of *k* (1 or 2), i.e., $\bar{k}=2$ when *k* = 1 and $\bar{k}=1$ when *k* = 2
- **H**_*b*_, ${\text{c}}_{b}\in {\mathbb{R}}^{6}$: inertia matrix and non-linear velocity-dependent term of the robot base attitude
- ${}^{k}{\text{H}}_{m}$, ${}^{k}{\text{c}}_{m}\in {\mathbb{R}}^{{}^{k}n}$: inertia matrix and non-linear velocity-dependent term of the robot arm *k*
- ${}^{k}{\text{H}}_{bm}\in {\mathbb{R}}^{6\times^{k}n}$: interference matrix between the robot base and robot arm *k*
- ${\text{x}}_{b}\in {\mathbb{R}}^{6}$: position and attitude of the robot base
- ^*k*^**ϕ**∈ℝ^*k*^*n*: joint angle of the arm *k*
- ^*k*^**τ**∈ℝ^*k*^*n*: joint torque of the arm *k*
- **F**_*b*_, ${}^{k}{\text{F}}_{h}\in {\mathbb{R}}^{6}$: external force and moment on the robot base and the end-effector of the arm *k*
- ${}^{k}{\text{J}}_{b}\in {\mathbb{R}}^{6\times^{k}n}$: Jacobian matrix with respect to the robot base and the robot arm *k*
- ${}^{k}{\text{J}}_{m}\in {\mathbb{R}}^{6\times^{k}n}$: Jacobian matrix with respect to the robot arm *k*

Thus, the equation of motion of the arm *k* in the joint space is derived as

(2) $${}^{k}\tau {=}^{k}H_{k}^{*}{\;}^{k}\overset{..}{\phi }{+}^{\bar{k}}H_{\bar{k}}^{*}{\;}^{\bar{k}}\overset{..}{\phi }{+}^{k}c^{*}{-}^{k}J_{k}^{{*}^{T}}{\;}^{k}F_{h}{-}^{\bar{k}}J_{k}^{{*}^{T}}{\;}^{k}F_{h}$$

where,

(3) $$\{\begin{array}{l} {}^{k}H_{k}^{*}{=}^{k}H_{m}{-}^{k}H_{bm}^{T}\;H_{b}^{-1}{\;}^{k}H_{bm}\\ {}^{k}c^{*}{=}^{k}c_{m}{-}^{k}H_{bm}^{T}\;H_{b}^{-1}c_{b}\\ {}^{k}J_{k}^{*}{=}^{k}J_{m}{-}^{k}J_{b}\;H_{b}^{-1}{\;}^{k}H_{bm}\\ {}^{k}J_{\bar{k}}^{*}={-}^{k}J_{b}\;H_{b}^{-1}{\;}^{\bar{k}}H_{bm} \end{array}$$

### 2.4. Contact model

The contact force and torque are generated at the contact point, where these are nominally defined as the force and torque acting on the target. That is, the force and torque in an inverse direction reacts on the end-effector of the chaser. In this paper, the contact force and torque acting on the target contact surface are converted into the force and torque acting on the center of mass position of the target, *A*. Given that the contact force ***F***_*P*_ exerts at a point *P* on the target surface, the converted force ***F***_*A*_ and torque ***T***_*A*_ acting on *A* are given as

(4) $$\begin{array}{rcl} {\text{F}}_{A} & = & {\text{F}}_{P} \end{array}$$

(5) $$\begin{array}{rcl} {\text{T}}_{A} & = & {\text{r}}_{AP}\times {\text{F}}_{P} \end{array}$$

where, ***r***_*AP*_ is the position vector of the contact point *P* from *A*.

**Figure 3:**
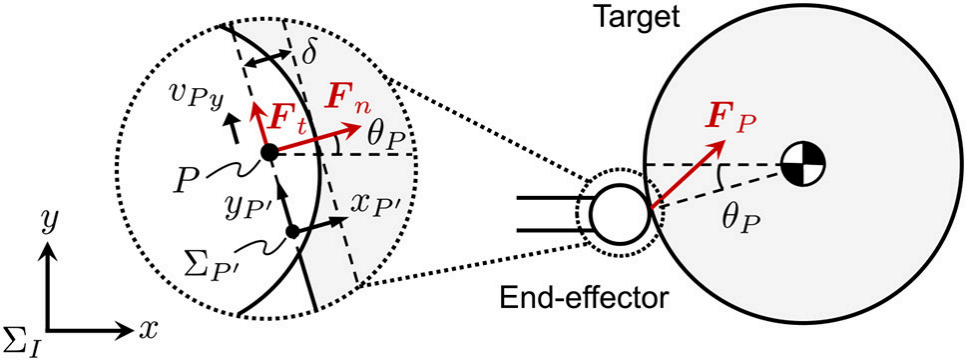
Contact force model of a spherical end-effector and a rigid target.

The contact force of a rigid body collision is typically approximated as a linear spring-damper model by a function of virtual penetration and its velocity (Gilardi and Sharf, 2002). In this paper, the contact forces are defined in the normal and tangential directions to the contact surface. In particular, the tangential contact force is modeled as a friction model. The contact force acting on the target, ***F***_*P*_, is defined as a resultant force of normal and tangential components, ***F***_*n*_ and ***F***_*t*_. Let $\underset{P}{\sum }$ be a contact coordinate system whose origin is the point *P*, where the *x*_*p*_-axis is oriented to the target center of mass and the *y*_*p*_-axis is tangential to the contact plane. Likewise, let $\underset{P^{\prime }}{\sum }$ be a coordinate system whose origin is a point *P*′ that is located near *P*, as shown in Figure 3. Here, $x_{P^{\prime }}$ and $y_{P^{\prime }}$ are parallel to *x*_*p*_ and *y*_*p*_, respectively. The unit vector of $x_{P^{\prime }}$ and $y_{P^{\prime }}$ are also ${\text{e}}_{P^{\prime }x}$ and ${\text{e}}_{P^{\prime }y}$, respectively.

The contact force normal to the contact surface is defined as a linear spring-damper model as

(6) $$F_{n}=\left(K_{n}\delta +C_{n}\dot{\delta }\right)e_{P^{\prime }x}$$

where *K*_*n*_ and *C*_*n*_ are the contact stiffness and damping coefficients in a normal direction to the contact surface, respectively.

In the tangential direction to the contact surface, we assume that kinetic friction is exerted during contact. The friction force is simply modeled as Coulomb friction. Given that *μ* and *v*_*Py*_ are, respectively, a kinetic frictional coefficient and relative tangential velocity on the contact surface, the tangential contact force can be given as

(7) $$\begin{array}{r} {\text{F}}_{t}=\text{sgn}\left(v_{Py}\right)\mu |{\text{F}}_{n}|{\text{e}}_{P^{\prime }y} \end{array}$$

where, sgn (·) is the signum function. In addition, the direction of the contact force is determined by *θ*_*P*_ regardless of the target attitude because the target is circular in shape. Consequently, ***F***_*P*_(***F***_*Px*_, ***F***_*Py*_) in Σ_*I*_ is written as

(8) $$\begin{array}{l} F_{Px}=|F_{n}|\left\{\cos\theta_{P}+\text{sgn}\left(v_{Py}\right)\mu \sin\theta_{P}\right\},\\ F_{Py}=|F_{n}|\left\{\sin\theta_{P}+\text{sgn}\left(v_{Py}\right)\mu \cos\theta_{P}\right\} \end{array}$$

## 3. Repeated impact-based control

This section elaborates on the control law in accordance with the models defined in the previous section. We propose a repeated impact-based control method for detumbling and capture of a spinning object by a dual-arm space robot in orbit. For the repeated impact, the dual-arm robot must control its dual arm to avoid escape of the target from its work-space. In this section, a tracking control law for the dual arm to adapt the post-impact target motion is first introduced. Then, a capture sequence of the target based on the repeated impact-based control is presented. Although the modeling in the previous sections is defined as three-dimensional as a generalized form, the following control law focuses simply on two-dimensional motion, as is assumed in section 2.

### 3.1. Motion tracking control

**Figure 4:**
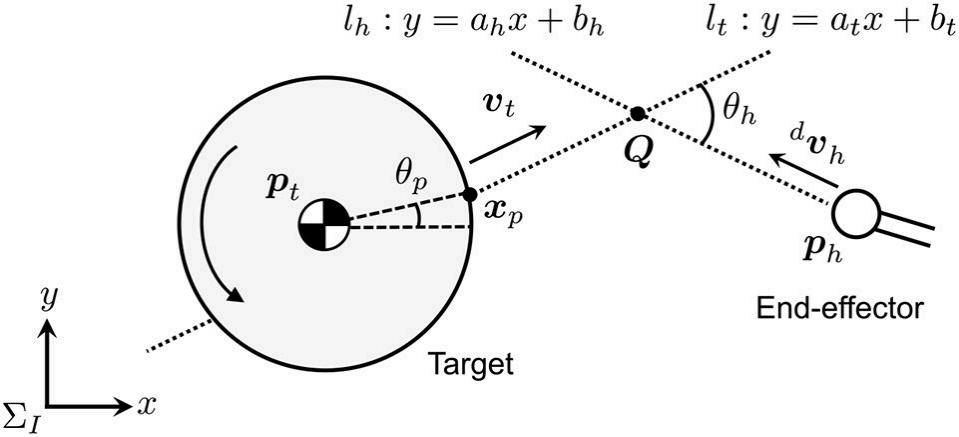
Path tracking control model for chaser arm.

Figure 4 illustrates the strategy of the motion tracking control for the dual arm to adapt the post-impact target motion. The dual-arm robot alternately contacts the target. In the proposed method, the target motion is predicted by remotely measuring/observing the position and velocity of its center of mass. Based on the sensing data, the robot arm is controlled to reach a confluence point ***Q*** earlier than the target in the tracking phase (Higashimori et al., 2007). During the motion tracking control of one arm, the other arm is de-actuated, and the attitude of the robot base is not controlled.

As the motion tracking law, the following procedure is introduced:

1. Set the contact angle *θ*_*p*_ and tracking angle *θ*_*h*_.
2. Calculate a motion path *l*_*t*_ of the target, which is predicted by the position and velocity of the center of mass, radius, and the contact angle *θ*_*p*_ of the target.
3. Calculate the confluence point ***Q*** of the arm and the target based on the arm's end-effector position, *l*_*t*_, and *θ*_*h*_.
4. Calculate the norm of the end-effector velocity so that it can reach ***Q*** earlier than the target.
5. Calculate the desired end-effector velocity ${}^{d}{\text{v}}_{h}$ by using *θ*_*h*_ and the norm of the end-effector velocity.

Here, ${\text{p}}_{t}={\begin{array}{cc} x_{t} & y_{t} \end{array}}^{T}$ and ${\text{v}}_{t}={\begin{array}{cc} {ẋ}_{t} & {ẏ}_{t} \end{array}}^{T}$ are, respectively, position and velocity of the center of mass of the target in Σ_*I*_, and the radius of the circular target is defined as *r*_*t*_. The position and velocity of the end-effector are also ${\text{p}}_{h}={\begin{array}{cc} x_{h} & y_{h} \end{array}}^{T}$,${\text{v}}_{h}={\begin{array}{cc} {ẋ}_{h} & {ẏ}_{h} \end{array}}^{T}$. Let the confluence point, the angle of the contact point from the target center of mass, and the angle between the predicted path and the desired end-effector's velocity be $\text{Q}={\begin{array}{cc} x_{Q} & y_{Q} \end{array}}^{T}$, *θ*_*p*_, and *θ*_*h*_, respectively. Here, the predicted motion path *l*_*t*_ is defined as *y* = *a*_*t*_*x* + *b*_*t*_.

From ***p***_*t*_ and ***v***_*t*_, *a*_*t*_ and *b*_*t*_ of *l*_*t*_ are given as follows:

(9) $$a_{t}=\frac{{\dot{y}}_{t}}{{\dot{x}}_{t}},\;b_{t}=\{\begin{array}{ll} -a_{t}\left(x_{t}+r_{t}\cos\theta_{p}\right)+\left(y_{t}+r_{t}\sin\theta_{p}\right) & :\;x_{h}>x_{t}\\ -a_{t}\left(x_{t}-r_{t}\cos\theta_{p}\right)+\left(y_{t}-r_{t}\sin\theta_{p}\right) & :\;x_{h}<x_{t} \end{array}$$

where ẋ_*t*_ ≠ 0 and ẏ_*t*_ ≠ 0. Likewise, given that the desired path of the arm is *l*_*h*_ : *y* = *a*_*h*_*x* + *b*_*h*_, the following relationship must be satisfied:

(10) $$\begin{array}{r} \tan\theta_{h}=\frac{a_{h}-a_{t}}{1+a_{h}a_{t}} \end{array}$$

Consequently, *a*_*h*_ and *b*_*h*_ are given as

(11) $$\begin{array}{r} a_{h}=\frac{a_{t}+\tan\theta_{h}}{1-a_{t}\tan\theta_{h}},\;b_{h}=-a_{h}x_{h}+y_{h} \end{array}$$

By solving *l*_*t*_ and *l*_*h*_, ***Q*** can be calculated as

(12) $$Q={\left[\begin{array}{cc} x_{Q} & y_{Q} \end{array}\right]}^{T}={\left[\begin{array}{cc} \frac{b_{h}-b_{t}}{a_{t}-a_{h}} & \frac{a_{t}b_{h}-a_{h}b_{t}}{a_{t}-a_{h}} \end{array}\right]}^{T}$$

To maintain the repeated impact, the arm needs to reach ***Q*** earlier than the target. Therefore, the following relation must be satisfied:

(13) $$\begin{array}{r} \frac{\left|\text{Q}-{\text{p}}_{h}\right|}{\left|{\text{v}}_{h}\right|}\leq \frac{\left|\text{Q}-{\text{x}}_{p}\right|}{\left|{\text{v}}_{t}\right|} \end{array}$$

Thus, the norm of the desired velocity of the end-effector, ${|}^{d}{\text{v}}_{h}{|}^{2}\left({={}^{d}{ẋ}_{h}}^{2}{+{}^{d}{ẏ}_{h}}^{2}\right)$, can be calculated as

(14) $$\begin{array}{r} {|}^{d}{\text{v}}_{h}|=k_{h}\frac{\left|\text{Q}-{\text{p}}_{h}\right|}{\left|\text{Q}-{\text{x}}_{p}\right|}|{\text{v}}_{t}| \end{array}$$

where, *k*_*h*_ is the control parameter and constant, which is determined so that the arm can reach ***Q*** earlier than the target. Thus, *k*_*h*_ ≥ 1 is given for the tracking control. Given the inclination angle of the desired path as *a*_*h*_ = ẏ_*h*_/ẋ_*h*_, the desired velocity of the end-effector is derived as

(15) $${}^{d}{\dot{x}}_{h}=\pm \frac{1}{\sqrt{1+a_{h}^{2}}}{\text{ |}}^{d}v_{h}\text{|},{\;}^{d}{\dot{y}}_{h}=\pm \frac{a_{h}}{\sqrt{1+a_{h}^{2}}}{\text{ |}}^{d}v_{h}\text{|}$$

The end-effector velocity is determined based on the relative position of the end-effector and the target center of mass. Hence, the end-effector's desired velocity ${}^{d}{\text{v}}_{h}$ can be given as

(16) $${}^{d}v_{h}=\{\begin{array}{ll} {\left[\frac{-1}{\sqrt{1+a_{h}^{2}}}\;{|}^{d}v_{h}|\;\frac{-a_{h}}{\sqrt{1+a_{h}^{2}}}\;|v_{h}|\;\right]}^{T} & :\;x_{h}>x_{t}\\ {\left[\frac{1}{\sqrt{1+a_{h}^{2}}}\;{|}^{d}v_{h}|\;\frac{a_{h}}{\sqrt{1+a_{h}^{2}}}\;{|}^{d}v_{h}|\;\right]}^{T} & :\;x_{h}<x_{t} \end{array}$$

Based on ${}^{d}{\text{v}}_{h}$, the desired joint velocity of the arm is introduced. The angular velocity of the end-effector while the arm is tracking the target, ${}^{d}{\text{ω}}_{h}^{k}$, is controlled to be zero so that the attitude of the end-effector is kept in the tracking mode. Moreover, the other arm stops its motion, and thereby its velocity is given as ***0***. Given ${\dot{x}}_{h}={\left[\begin{array}{cc} {}^{1}{\dot{x}}_{h}^{T} & {}^{2}{\dot{x}}_{h}^{T} \end{array}\right]}^{T}$, the desired joint angle of the dual-arm, ${}^{d}\overset{{}^{\circ}}{\text{ϕ}}$, can be calculated as follows.

(17) $${}^{d}\dot{\phi }=J^{+}{\;}^{d}{\dot{x}}_{h}$$

where, ***J***^+^ is the pseudo-inverse matrix of the generalized Jacobian matrix (Umetani and Yoshida, 1993).

### 3.2. Control parameters

**Figure 5:**
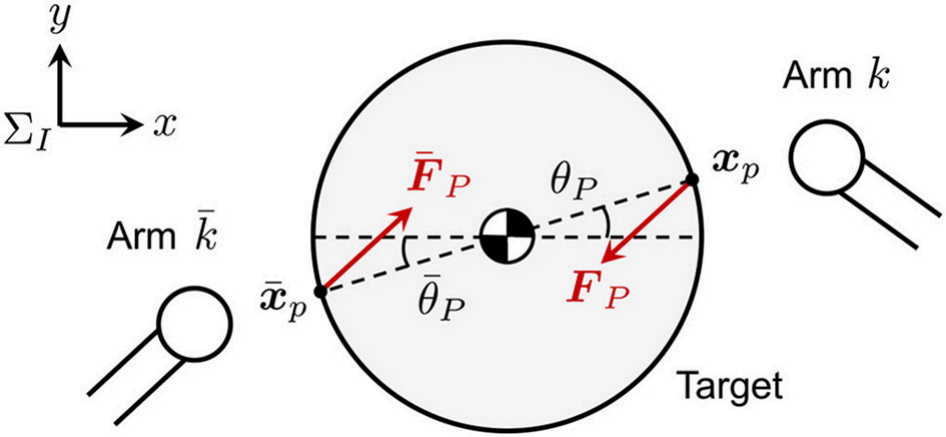
Schematic of relationship between contact force and contact point.

To maintain the repeated impact, selection of the contact point on the target surface is a key parameter. For instance, assuming the desired contact points on a frictional object, ***x***_*p*_ and ${\bar{\text{x}}}_{p}$, are independently defined as *θ*_*p*_ and ${\bar{\theta }}_{p}$, as shown in Figure 5, the direction of the target motion and desired contact point are changed by each impact because the resultant contact force ***F*_*P*_** is not oriented to the target center of mass. In this case, the arm is at risk of not tracking the target motion. Therefore, the control constraint $\theta_{p}={\bar{\theta }}_{p}$ is given so that the angle of the contact point becomes constant. Based on the contact model, for the case where *μ* = |***F***_*t*_|/|***F***_*n*_| is given as a constant value, the direction of the contact force is also constant even if the force amplitude varies. Hence, the constraint $\theta_{p}={\bar{\theta }}_{p}$ allows the restraint of the target motion in a single-axial direction in Σ_*I*_.

Let the directional angle of the velocity of the end-effector to the target's motion path *l*_*t*_ be *θ*_*h*_. Given 0 ≤ *θ*_*h*_ ≤ π/2, the dual-arm is controlled to simultaneously track the given path and close the gap distance between the end-effectors. In the proposed method, the symmetric control parameter $\theta_{h}={\bar{\theta }}_{h}$ is applied as a typical case.

### 3.3. Stable capture condition

The final state of the motion tracking control corresponds to the capture completion of the target by the dual arm. This shows that detumbling and capture can be achieved by the common control framework. When $\theta_{h}={\bar{\theta }}_{h}$ is satisfied, the contact points and the target center of mass are located along a straight line because each arm targets its desired contact point as ***x***_*p*_ or ${\bar{\text{x}}}_{p}$. Therefore, the stable capture state can be achieved, by which the velocity of both end-effectors is controlled to be zero at the final state, where they simultaneously contact the target.

### 3.4. Capture sequence

**Figure 6:**
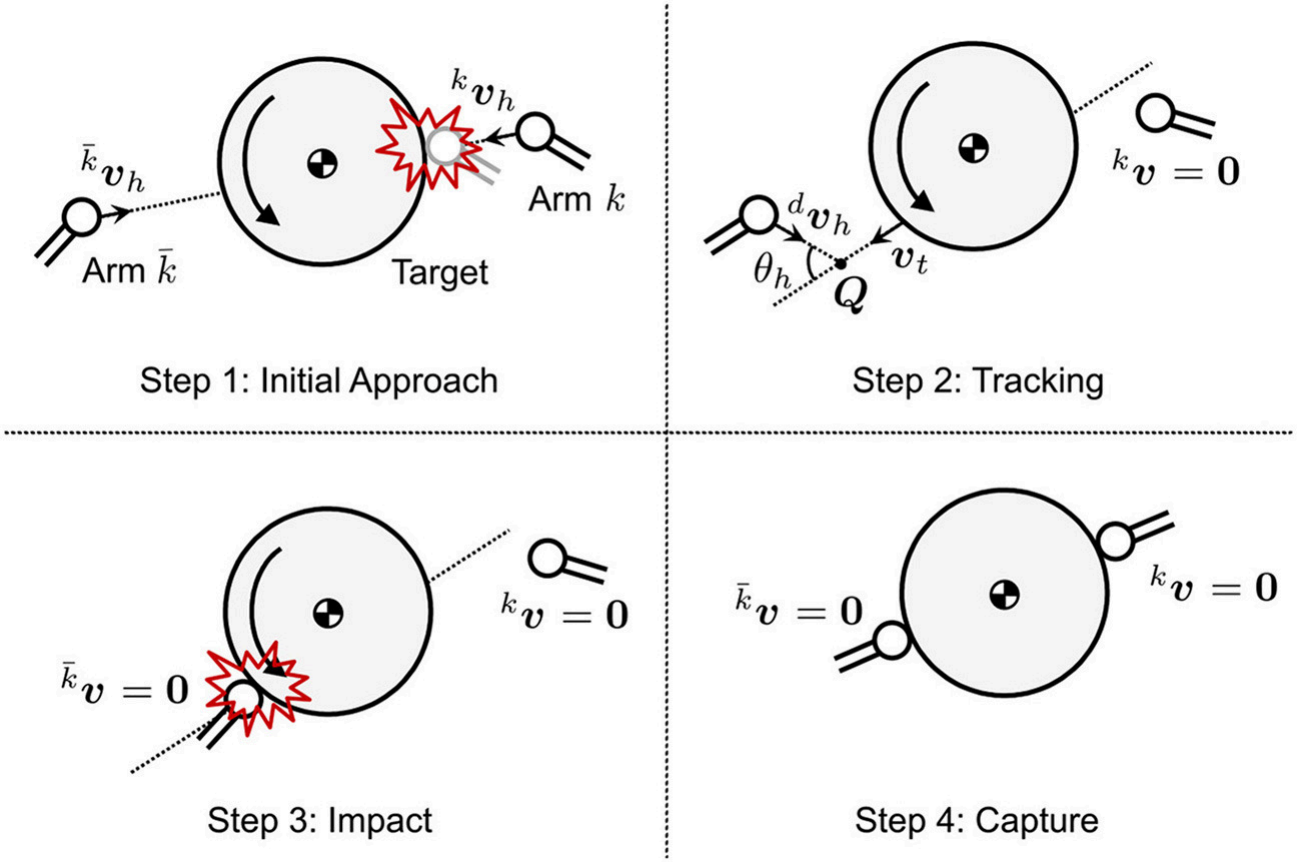
Capture sequence based on repeated impact by a dual-arm space robot.

Figure 6 shows a schematic view of the repeated impact-based capture sequence of the free-floating target by the dual-arm space robot. The sequence is summarized in the following four steps, where it is assumed that the post-approaching state after the dual-arm robot is controlled to approach the target, so that the relative linear velocity becomes zero.

1. The dual-arm is controlled to approach the target with a constant velocity ${}^{k}{\text{v}}_{h}\left({=}^{\bar{k}}{\text{v}}_{h}\right)$ until one of the dual-arm shoves the target away.
2. After the impact, the other arm ahead of the target motion is controlled by the path tracking control with ${}^{d}{\text{v}}_{h}$ to the target velocity ***v***_*t*_ before the impact. Simultaneously, the other arm maintains a stationary state.
3. During the impact, both arms maintain all the joint angles. Through the impact, the linear and angular velocity of the target obviously decay.
4. The robot repeats sequences 2 and 3 until both arms contact the target simultaneously (i.e., the state where the capture is completed).

The proposed method enables the detumbling and capture of the target by a single control law. Moreover, this method can be applied to the capture of uncertain debris because the precise values of the target's inertial properties and surface physics are explicitly not included. Although the control method targets the planar motion to discuss its fundamental effectiveness and feasibility for starters, its basic concept can be expanded to a three-dimensional situation.

## 4. Simulation analysis

Based on the proposed control method, this section presents a two-dimensional simulation analysis. The numerical simulation shows the validity and effectiveness of the proposed method.

### 4.1. Chaser and target model

**Figure 7:**
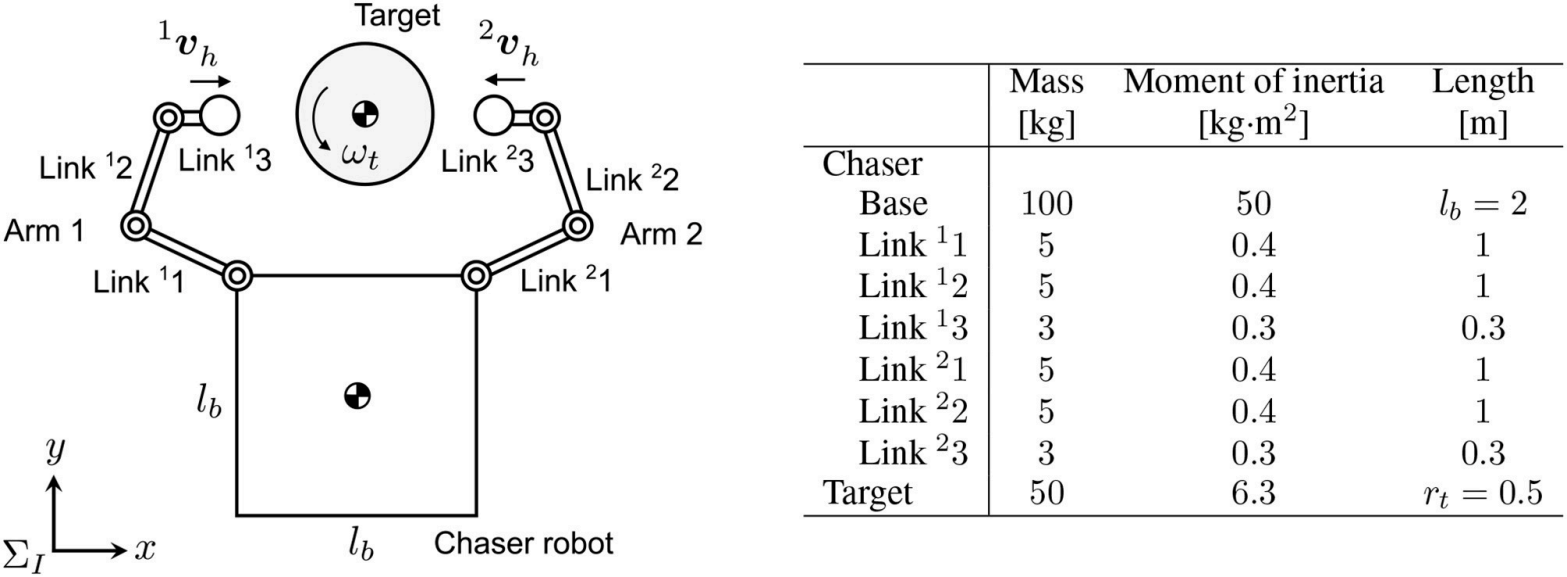
Simulation model of chaser robot and target (left: schematic view, right: link parameters).

The chaser model is a free-flying robot that has dual-arms with three-DOF joints on each. The two-dimensional target model is simply assumed to be a cylindrical rigid object whose center of mass is located on its geometric center. Figure 7 shows a schematic view of the models and their parameters that are used in the subsequent simulations. The end-effector of the arm was modeled as a rigid sphere whose diameter of 0.2 m. The mechanical compliant wrist (Uyama et al., 2012), specifically, a helical spring, is also assumed to be embedded in the arm. Basically, the spring stiffness is designed to be much lower than the contact stiffness of a rigid body collision. Employing this configuration enables the approximation of the contact parameters as known mechanical properties of the spring of *K*_*n*_ = 900 [N/m] and *C*_*n*_ = 6 [N·s/m] in the contact force model of the rigid body collision. Such a compliant component also works passively as an adaptive factor to errors of sensing the relative position/motion and controlling the end-effector. Moreover, *μ* = 0.1 is set as an unknown parameter for the chaser.

### 4.2. Simulation conditions

The initial angular velocity of the target is ω_*t*0_, and the initial approaching velocities of the arm are ${}^{1}{\text{v}}_{h}$ and ${}^{2}{\text{v}}_{h}$. Also, both the initial linear velocity of the relative motion and the initial angular velocity of the chaser are set to zero. To simplify the problem, the end-effectors are initially located on a straight line that passes through the target center of mass.

In the simulation analysis, we set ^1^***ϕ*** = [60° −90°− 60°]^*T*^, ^2^***ϕ*** = −60° 90° 60°^*T*^, ${}^{1}{\text{v}}_{h}={\begin{array}{cc} 0.05 & 0 \end{array}}^{T}$ [m/s], ${}^{2}{\text{v}}_{h}={\begin{array}{cc} -0.05 & 0 \end{array}}^{T}$ [m/s], ${\text{v}}_{t}={\begin{array}{cc} 0 & 0 \end{array}}^{T}$ [m/s], and ω_*t*0_ = 10 [°/s]. In addition, $\theta_{P}=0^{{}^{\circ}}$, $\theta_{h}=40^{{}^{\circ}}\left(<90^{{}^{\circ}}\right)$, and *k*_*h*_ = 10 are used as the control parameters.

### 4.3. Results and discussion

**Figure 8:**
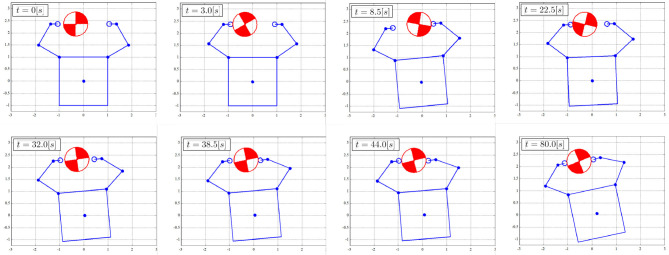
Snapshots of simulation result.

**Figure 9:**
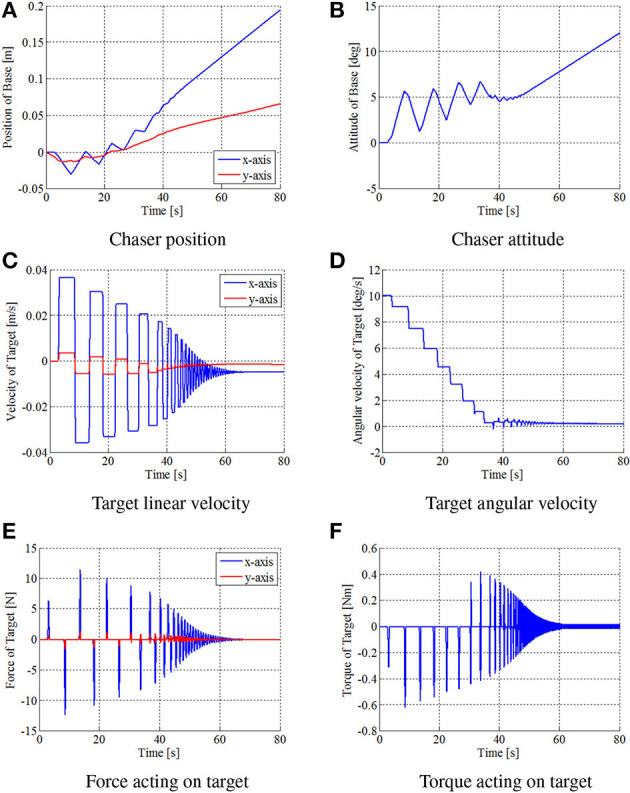
Time histories of simulation variables in simulation. **(A)** Chaser position. **(B)** Chaser attitude. **(C)** Target linear velocity. **(D)** Target angular velocity. **(E)** Force acting on target. **(F)** Torque acting on target.

**Figure 10:**
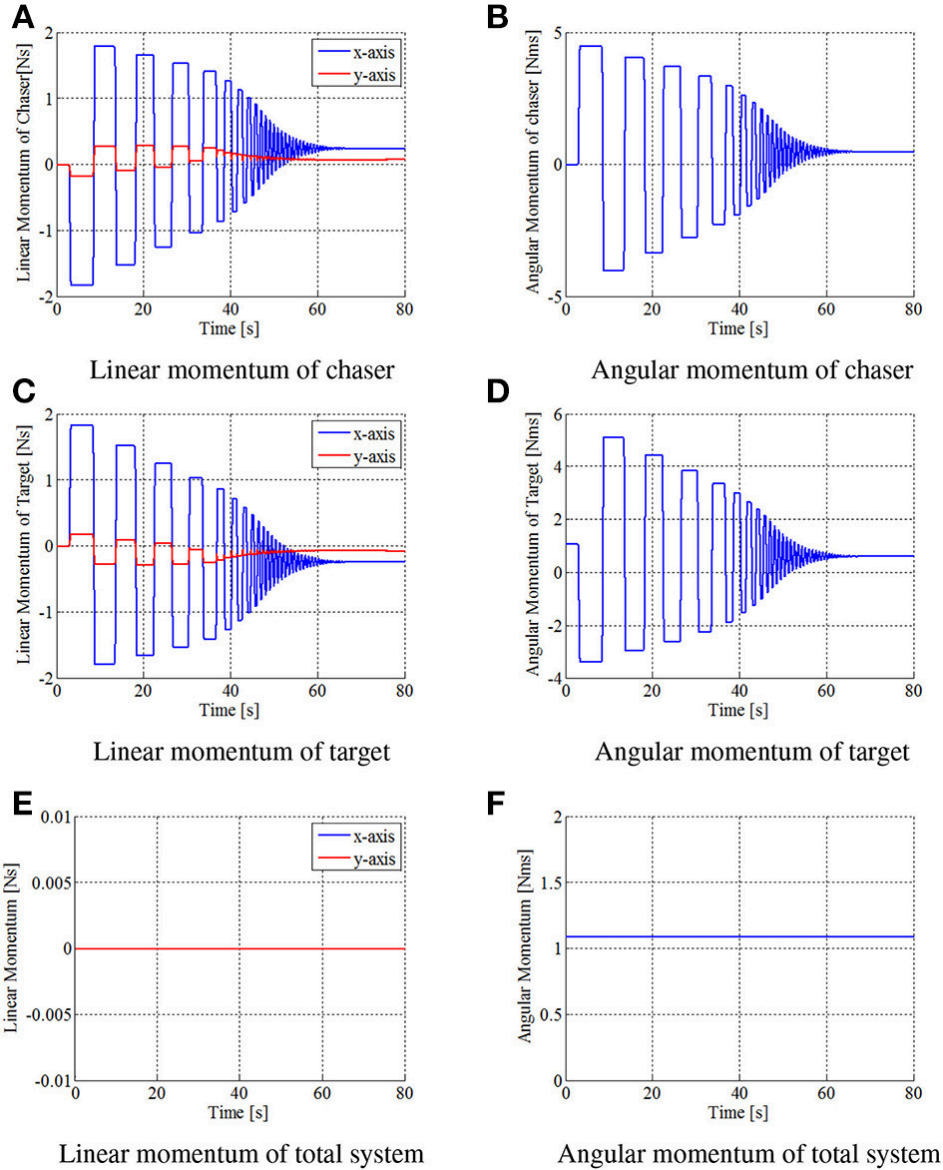
Time histories of momentum in simulation. **(A)** Linear momentum of chaser. **(B)** Angular momentum of chaser. **(C)** Linear momentum of target. **(D)** Angular momentum of target. **(E)** Linear momentum of total system. **(F)** Angular momentum of total system.

Figures 8–10 show the simulation results. Figure 8 shows snapshots of the chaser and target, and Figures 9, 10 depict the time histories of the variables. The plotted variables are represented in the inertial coordinate system, except for the desired contact position and the distance between the end-effectors.

The results confirm that the repeated impulse-based capture is achieved. The stable capture state is almost completed at *t* = 44 [s]. From Figures 9, 10, the linear and angular velocity of the target is damped by each impact. Consequently, the result showed that the target velocity converged. After *t* = 40 [s], the rate of damping of the velocity, force, and momentum become exponentially larger. This is because both end-effectors just contacted the target, and the damping effect thereby increased. The result also shows that the chaser and the target have a constant angular velocity in the post-capture state. Throughout the simulation, conservation of momentum of the whole system is satisfied, as shown in Figures 10E, F. Although the capture performance is intricately linked with various parameters of both the robot and target, it is concluded that the effectiveness of the capture of an uncertain target based on the proposed control method is numerically verified.

## 5. Experiment

Following the simulation analysis, this section presents the experimental evaluation to verify the fundamental feasibility of the proposed control method.

### 5.1. Experimental setup

**Figure 11:**
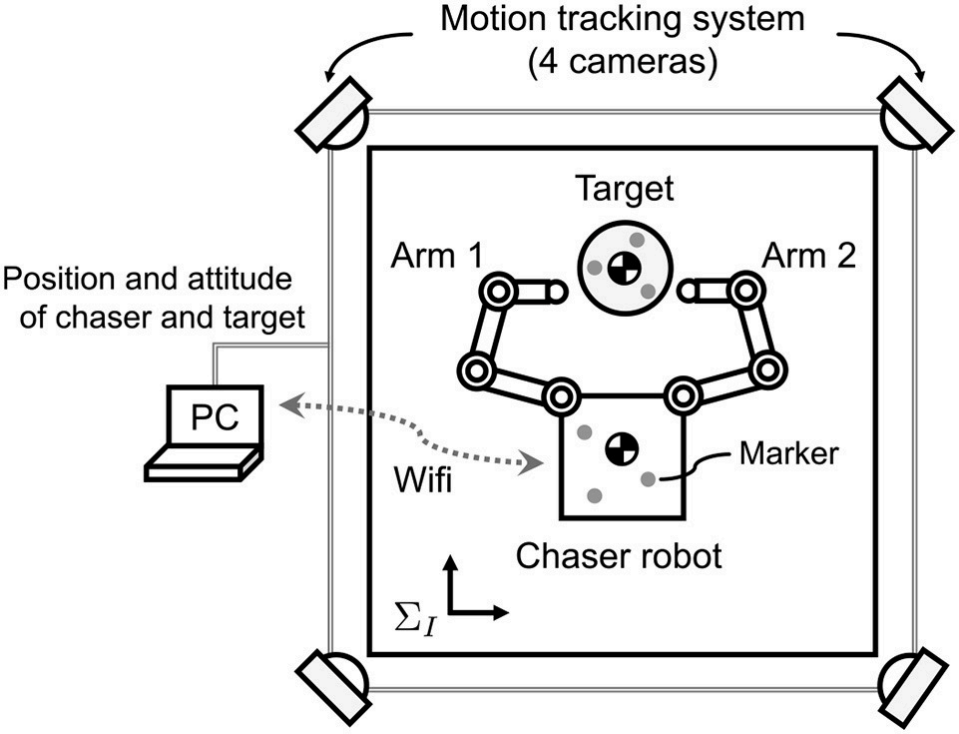
Experimental setup (top view).

Figure 11 shows an overview of the experimental setup. Air floating test beds that can emulate planar motion in microgravity on a flat stone plane were used for simulating the chaser robot and target. Through the experiment, the position and attitude of the chaser base and target were precisely measured by an external motion tracking camera system (OptiTrack FLEX:V100R2; NaturalPoint Inc.) whose sampling frequency is 100 Hz. The tracking data was transmitted to the chaser as feedback information without any cables. Thus, using an on-board computer, the chaser was able to calculate the position and linear velocity of the target center of mass, as well as the attitude angle and angular velocity around it.

### 5.2. Air floating test bed

**Figure 12:**
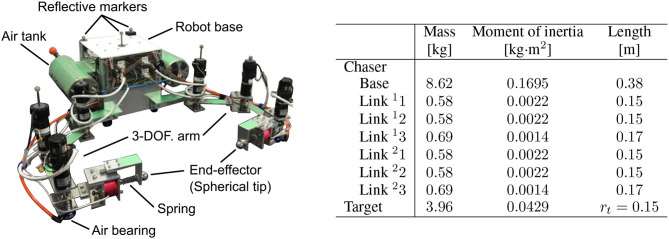
Air floating test beds for planar microgravity experiment (left: overview of a chaser test bed of a dual-arm robot, right: link parameters).

In the experiment, two air floating test beds were used as the dual-arm chaser robot and the cylindrical target. These test beds were equipped with air-tanks and air-bearings (S102501 and S104001; NEWWAY Air Bearings) on their bottom surface. Pressure-controlled air-injection from the air-bearings enabled the test beds to perform frictionless motion for several minutes. Figure 12 shows an overview of the air floating robot. The robot has a three-DOF dual-arm system, where an aluminum spherical tip and a spring as mechanical compliance are attached on each arm. The air floating target quips an acrylic pipe with plastic tapes on its surface to emulate a circular object in two dimensions.

In the robot base, an on-board computer (NANO-8050; Portwell, Inc.), micro-controllers (SH7125 and SH7144; Renesas Electronics Corp.), and motor drivers (1XH Power Module; HiBot Corp.) for driving the dual-arm are installed. Lithium-ion batteries (E-HL9S; IDX Company, Ltd.)are mounted on the robot base as the power source. The chaser robot can control its motion based on the on-board computer. Additionally, the real-time data of the position and attitude of the robot base and target obtained by the motion tracking cameras can be sent to the on-board computer via wireless communication. The arm comprises three DC brushed motors (RH-8D-3006-E100AL; Harmonic Drive Systems, Inc.) and incremental encoders on each.

### 5.3. Experimental conditions

As the initial relative states, the linear velocity of the chaser and target was set to zero, and only the target angular velocity was given. In the experiment, we also set ^1^***ϕ*** = [60° −40° −110°]^*T*^, ^2^***ϕ*** = −60° 40° 110°]^*T*^, ${}^{1}{\text{v}}_{h}={\begin{array}{cc} 0.015 & 0 \end{array}}^{T}$ [m/s], ${}^{2}{\text{v}}_{h}={\begin{array}{cc} -0.015 & 0 \end{array}}^{T}$ [m/s], $\theta_{P}=0^{{}^{\circ}}$, $\theta_{h}=60^{{}^{\circ}}$, and *k*_*h*_ = 6. The control parameters were selected with the same values as in the previous simulation. Furthermore, the initial center of mass position of the target was also located between the end-effectors with an offset distance.

### 5.4. Results and discussion

**Figure 13:**
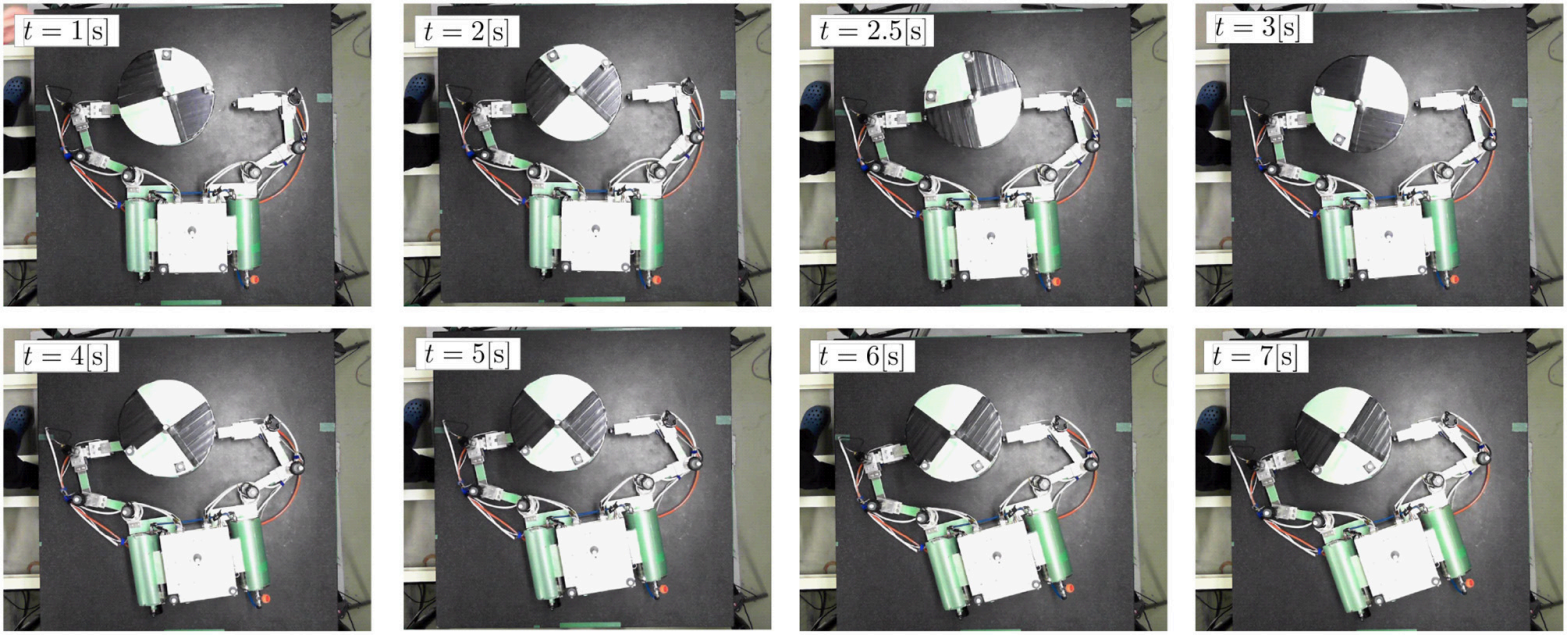
Snapshots of experimental result (top view).

**Figure 14:**
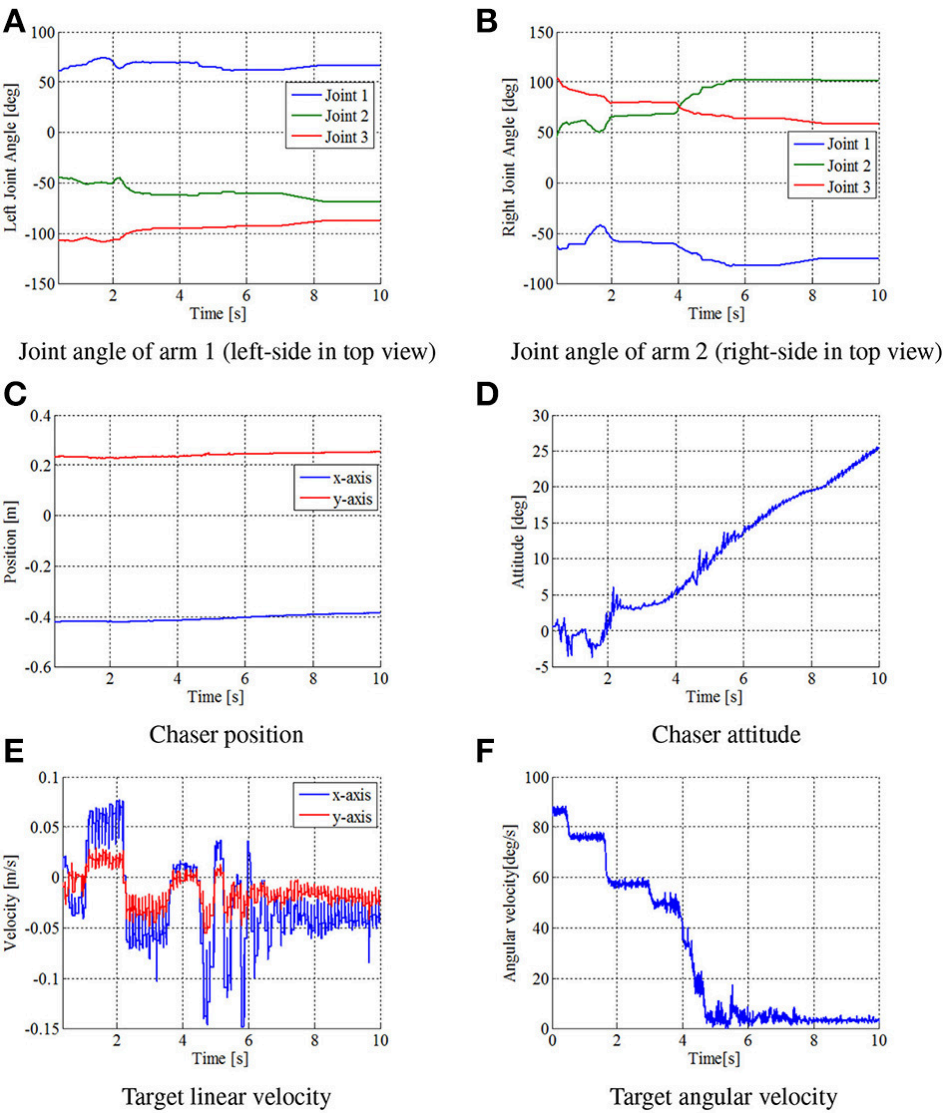
Time histories of state variables in experiment. **(A)** Joint angle of arm 1 (left-side in top view). **(B)** Joint angle of arm 2 (right-side in top view). **(C)** Chaser position. **(D)** Chaser attitude. **(E)** Target linear velocity. **(F)** Target angular velocity.

Figures 13, 14 show the experimental results. Figure 13 shows snapshots of the top view of the experiment. Figure 14 depicts the time histories of the sensor data.

First, the results confirmed that the repeated impulse-based capture was experimentally demonstrated. From Figures 14A,B, the time histories of the joint angles show the change of the motion tracking control. Although the change of the target linear velocity was relatively small because of a small damping coefficient of the spring, the target angular velocity was damped at each impact. After *t* = 6 [s], the target angular velocity was suppressed by the frictional effect, as is the case in the simulation. It is also confirmed that the motion of the chaser and target converged to a steady state with a small constant velocity in the final capture state. Accordingly, the results conclude that fundamental feasibility of the capture of an uncertain target based on the proposed control method is experimentally demonstrated.

To apply the proposed capture method to more complicated debris like a malfunctioning satellite with tumbling motion in three-dimension, effects of the debris' shape and surface roughness and tumbling motion will need to be considered. Furthermore, to cope with such a challenging capture mission, the tracking control law must be improved in addition to higher DOF of the arm. The current tracking control assumes that the debris' shape is axially symmetric, and thereby there is little constraint on the contact timing and the arm motion so far as the end-effector reaches a confluence point faster than the target. Thus, the improvement of the tracking control, which proper momentum exchange is repeatedly accomplished, a possible future work for advanced applications.

## 6. Conclusion

This paper presented a repeated impact-based capture control for a dual-arm space robot and its experimental validation. As the initial study of the repeated impact-based capture, the capture target was assumed to be a rocket upper stage that can be modeled as a single spinning cylinder. The proposed control method can achieve detumbling and capture of spinning space debris having uncertainties in its mass and moment of inertia. The validity of the control method was also demonstrated based on the numerical and experimental analyses. In particular, a key contribution of this paper is the verification of the fundamental feasibility of the control method through an experimental evaluation on the ground, while the target is simply assumed to be a cylindrical object. Hence, the results of this study are expected to contribute to a real space robot system. As the next step of this study, an implementation of on-board sensing capable of measuring the relative position and velocity, in addition to a robustness analysis of the developed control method to the initial conditions and control parameters, will be addressed. Furthermore, the method needs to be expanded to three-dimensional capture and a more complicated target shape and surface roughness like a malfunctioning satellite.

## Author contributions

KN, RK, and KY conceived of the presented idea. KN and RK developed the theoretical framework and performed the experimental verification. KY supervised this work. All authors discussed the results and contributed to the final manuscript.

### Conflict of interest statement

The authors declare that the research was conducted in the absence of any commercial or financial relationships that could be construed as a potential conflict of interest.
